# Integrative multi‐omics analysis reveals the critical role of the *PBXIP1* gene in Alzheimer's disease

**DOI:** 10.1111/acel.14044

**Published:** 2023-11-20

**Authors:** Jingyun Zhang, Xiaoyi Sun, Xueqing Jia, Binggui Sun, Shijun Xu, Weiping Zhang, Zuyun Liu

**Affiliations:** ^1^ Center for Clinical Big Data and Analytics of the Second Affiliated Hospital, and Department of Big Data in Health Science School of Public Health, the Key Laboratory of Intelligent Preventive Medicine of Zhejiang Province Zhejiang University School of Medicine Hangzhou Zhejiang China; ^2^ Department of Neurobiology, School of Basic Medical Sciences, Key Laboratory of Medical Neurobiology (Ministry of Health of China), Key Laboratory of Neurobiology of Zhejiang Province Zhejiang University School of Medicine Hangzhou Zhejiang China; ^3^ Institute of Material Medica Integration and Transformation for Brain Disorders, and School of Pharmacy Chengdu University of Traditional Chinese Medicine Chengdu Sichuan China; ^4^ Department of Pharmacology, Institute of Neuroscience, Key Laboratory of Medical Neurobiology of the Ministry of Health of China, Zhejiang Province Key Laboratory of Mental Disorder's Management Zhejiang University School of Medicine Hangzhou Zhejiang China

**Keywords:** aging, Alzheimer's disease, DNA methylation clocks, neuropathology, proteomics, RNA‐seq

## Abstract

Alzheimer's disease (AD) is a neurodegenerative disorder, and its strongest risk factor is aging. A few studies have explored the relationship between aging and AD, while the underlying mechanism remains unclear. We assembled data across multi‐omics (i.e., epigenetics, transcriptomics, and proteomics, based on frozen tissues from the dorsolateral prefrontal cortex) and neuropathological and clinical traits from the Religious Orders Study and Rush Memory and Aging Project (ROSMAP). Aging was assessed using six DNA methylation clocks (including the Horvath clock, Hannum clock, Levine clock, HorvathSkin clock, Lin clock, and Cortical clock) that capture mortality risk in literature. After accounting for age, we first identified a gene module (including 263 genes) that was related to the integrated aging measure of six clocks, as well as three neuropathological traits of AD (i.e., β‐amyloid, Tau tangles, and tangle density). Interestingly, among 20 key genes with top intramodular connectivity of the module, *PBXIP1* was the only one that was significantly associated with all three neuropathological traits of AD at the protein level after Bonferroni correction. Furthermore, *PBXIP1* was associated with the clinical diagnosis of AD in both ROSMAP and three independent datasets. Moreover, *PBXIP1* may be related to AD through its role in astrocytes and hippocampal neurons, and the mTOR pathway. The results suggest the critical role of *PBXIP1* in AD and support the potential and feasibility of using multi‐omics data to investigate mechanisms of complex diseases. However, more validations in different populations and experiments in vitro and in vivo are required in the future.

## Graphical abstract

Using multi‐omics data and AD's neuropathology from the ROSMAP study, we identified a gene module (m108) related to both aging (DNA methylation clocks) and AD. We also validated that PBXIP1, the hub gene of m108, was associated with AD's neuropathology at the proteomic level and AD's diagnosis in several independent datasets. Our study supported the potential of using multi‐omics data to investigate complex diseases and implied that PBXIP1 and related pathways might explain the mechanism of AD.

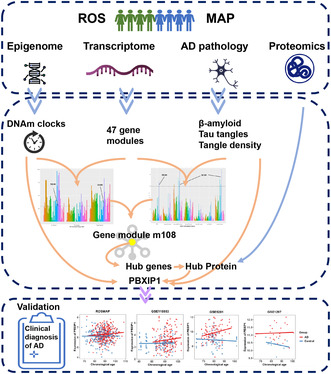

**Abbreviations**
AA: age acceleration
AD: Alzheimer's disease
CA: chronological age
DLPFC: dorsolateral prefrontal cortex
GEO: Gene Expression Omnibus
MAP: the Memory and Aging Project
PCA: Principal component analysis
ROS: The Religious Orders Study
ROSMAP: The Religious Orders Study and Rush Memory and Aging Project

## 1 INTRODUCTION

Alzheimer's disease (AD) is a neurodegenerative disorder characterized by gradual declines in cognitive functions and activities of daily living, simultaneously with many neuropsychiatric symptoms (Eikelboom et al., 2021; McKhann et al., 2011). The pathological characteristics of AD include the accumulation of amyloid‐β plaques and neurofibrillary tangles of hyperphosphorylated Tau in the brain (Hyman et al., 2012; Moscoso et al., 2021). As the leading cause of dementia, AD contributes to at least 2/3 of dementia in people aged 65 and older (Kumar et al., 2021), leading to heavy societal and personal burdens in an aging society. However, there is no cure for AD and only a few interventions could relieve some symptoms of AD (Kumar et al., 2021), requiring an urgent need of prevention and treatment strategy of AD.

As the strongest risk factor of AD (Hou et al., 2019), aging is a comprehensive and multi‐dimensional process and could be measured at different hierarchical levels, including biological, phenotypic, and functional levels (Ferrucci et al., 2018). Epigenetic clock or DNA methylation clock, as one of the biological aging measures, is a hotspot and is used widely in aging research. More than 10 DNA methylation (DNAm) clocks have been proposed and reported to be associated with age‐related diseases (Levine et al., 2015; Martin‐Herranz et al., 2019) and mortality (Chen et al., 2016; Marioni et al., 2015). Although a few studies have explored the association between DNAm clocks and AD (Grodstein et al., 2021) and AD pathological indicators (Grodstein et al., 2021; Levine et al., 2015), the underlying mechanism remains unclear.

Integrating analysis based on multi‐omics data provides an opportunity to understand complex diseases including AD. Based on a previous study by Mostafavi et al. (2018), Yu et al. (2018) identified 12 AD‐related proteins expressed at the dorsolateral prefrontal cortex (DLPFC) in a gene module m109 using targeted proteomics. These proteins might involve in AD via two molecular pathways: one should be amyloid deposition, and the other might be resilience without a known pathological footprint (Yu et al., 2018). Xie et al. (2021) proposed an AD disease status prediction model that integrated 305 multi‐omics features including 74 SNPs, 173 genes, and 58 proteins, which were mainly associated with amyloid deposition, cortical volume, and average thickness of frontal regions. These studies have showed that integrative multi‐omics analysis has the potential to investigate the molecular mechanism of AD.

**FIGURE 1:**
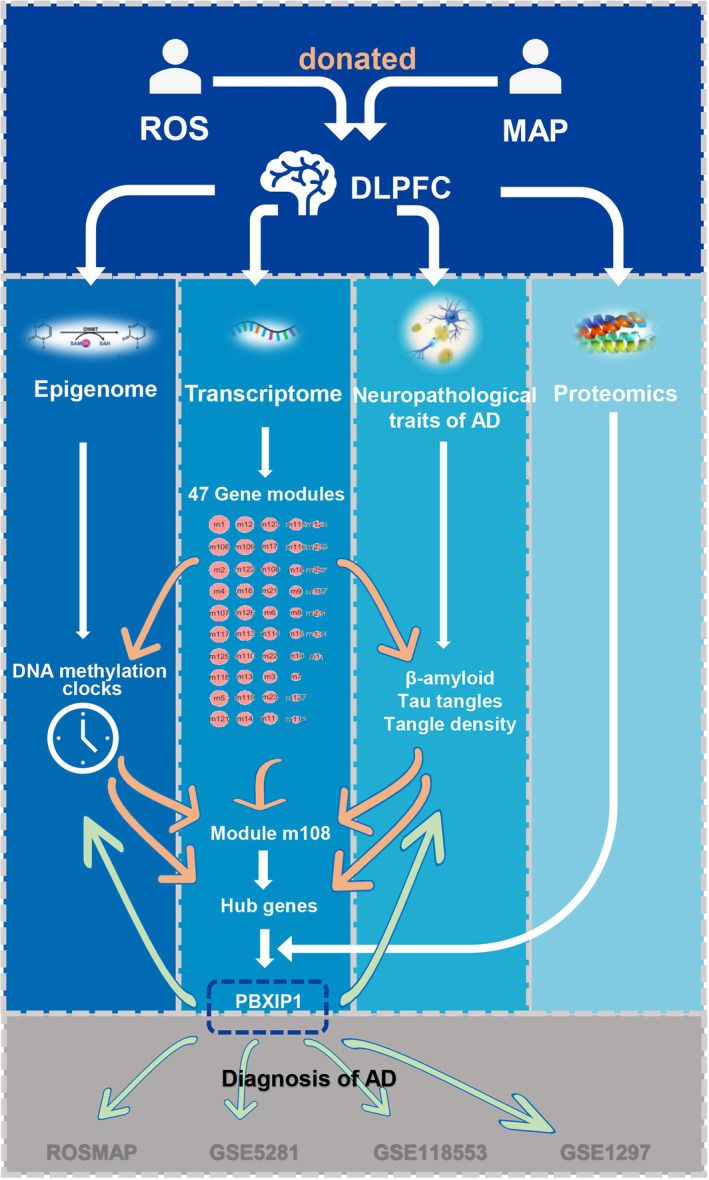
Roadmap for the integrative multi‐omics analysis. ROS, the Religious Orders Study; MAP, the Memory and Aging Project; DLPFC, dorsolateral prefrontal cortex; AD, Alzheimer's disease.

As shown in Figure 1, this study is based on the Religious Orders Study and Rush Memory and Aging Project (ROSMAP, mainly analytic sample involving multi‐omics data of DLPFC) and Gene Expression Omnibus (GEO). Instead of identifying candidate DNAm cites of AD in previous studies based on RSOMAP (Huo et al., 2019; Wang et al., 2020), we considered DNAm clocks (a combination of many preselected DNAm cites) as powerful aging measures and investigated candidate genes or proteins that were related to both aging and the neuropathological traits of AD. More specifically, we aimed to (1) identify the optimal DNAm clocks which capture neuropathological traits of AD; (2) conduct a transcriptome network analysis based on optimal DNAm clocks and AD pathologies to identify key gene modules and hub genes; (3) investigate the key proteins closely related to key gene modules and hub genes based on proteomics; and (4) further examine the association between hub genes, key gene modules and neuropathological traits of AD.

## 2 MATERIALS AND METHODS

### 2.1 Participants

The ROSMAP is composed of two community‐based longitudinal, epidemiologic clinical‐pathologic cohort studies of aging and dementia (Bennett et al., 2005, 2012). Religious Orders Study (ROS), beginning in 1994, enrolled participants at least 55 years old without known signs of dementia from religious communities for longitudinal clinical examination and brain donation. In 1997, the Memory and Aging Project (MAP) began, which complemented ROS by enrolling participants with broader life experiences and socioeconomic status, having a similar research design and structure to ROS. Both cohorts are managed by Rush University, and all procedures strictly follow the specifications listed in the pathology dataset recommended by the National Alzheimer's Disease Coordinating Center.

This study used data across multi‐omics (i.e., DNA methylation, RNA‐seq, proteomics, based on frozen tissue from DLPFC donated by ROSMAP participants after death) and neuropathological traits of 761 participants. DLPFC is an area involved in AD pathology during the late stages of the disease (Casula et al., 2023), and is reported to be important in maintaining mental representations in working memory. There is a near perfect correspondence between the cortical hierarchy and the pattern and sequence of tau pathology (Arnsten et al., 2021). All data were obtained through the AMP‐AD Knowledge Portal and Rush Alzheimer's Disease Center of Rush University (https://adknowledgeportal.synapse.org/Explore/Programs/DetailsPage?Program=AMP‐AD).

### 2.2 DNA methylation and DNAm clocks

DNA was extracted from frozen DLPFC sections of 761 deceased participants, and DNA methylation data were sequenced by the Illumina InfiniumHumanMethylation450 bead chip assay. Quality control of these data had been pre‐conducted, including excluding low‐quality probes and samples and correction of batch effects (De Jager et al., 2014).

According to the previous studies (Liu et al., 2020; Lu et al., 2019; Shireby et al., 2020), 13 DNAm clocks were calculated, and *z*‐score‐transformed for further comparison. Except for Cortical age and GrimAge, we used the first author's family name to refer to each clock. Most of these clocks are used to predict chronological age (CA) in whole blood (except for Harvoth, HarvothSkin, and Cortical clocks), and the number of CpG included ranges from 3 to 1030 (only Bocklandt and Garagnani clocks are based on one CpG). Besides, age acceleration (AA), which was defined as the residual of the linear model when regressing the DNAm clock on CA, was also calculated. AA reflected the paces of aging of individuals, tissues, or cells, that is, whether an individual, tissue, or cell is older (positive value) or younger (negative value) than expected.

### 2.3 RNA sequence (RNA‐seq)

The RNA‐seq data of ROSMAP was sequenced from the gray matter of DLPFC of 542 samples by Next‐generation RNA sequencing (the Illumina HiSeq with 101 bp paired‐end reads) (Mostafavi et al., 2018). These RNA‐seq data had completed the preliminary quality, including Fragments Per Kilobase Million standardization, normalization, and correction of batch effects. Then two quality control steps were conducted. First, we estimated the associations between each sample and all other samples based on gene expression profiles to remove outlier samples. By defining the average association between sample i and other samples as Di, a total of nine samples were considered outliers and excluded due to Di <0.9 (Hebert et al., 2013). Second, the ClusterProfiler package and biomaRt package of R language were used to convert Ensembl ID to Gene Symbol, and then pseudogenes were excluded. The average value of the expression with the same Gene Symbol remained as the actual expression of the gene. Low‐expressed genes with expressions <1.0 were filtered out. Then, after the quality control steps above, the expressions of genes were log2‐transformed. At last, the genes were clustered into 47 modules based on the weighted gene co‐expression network analysis (WGCNA) constructed by Mostafavi et al. (2018).

### 2.4 Proteomics

The tandem mass tag (TMT) isobaric labeling mass spectrometry method was used to measure the protein abundance from cortical microdissections of the DLPFC of ROSMAP subjects (Wingo et al., 2020). Four hundred samples were allocated randomly to 50 batches based on age, sex, postmortem interval (PMI), diagnosis, and neuropathological traits. MS/MS (MS2) and SPS‐MS3 techniques were used for 45 and five TMT batches via the Orbitrap Fusion mass spectrometer (Thermo Fisher Scientific), respectively. The results have already been normalized and log2‐transformed (https://www.radc.rush.edu/). For proteins with the same official gene symbols, we chose the largest abundance, and the missing data were imputed by K Nearest Neighbors methods.

### 2.5 Neuropathological traits of AD

Brain autopsy followed standard protocol (Schneider et al., 2009). In this study, we mainly considered three indicators: β‐amyloid protein level, tangle density, and neurofibrillary tangle summary.

The β‐amyloid protein level was identified by molecularly‐specific immunohistochemistry and quantified by image analysis. Its value was calculated as the average percentage of cortex area occupied by β‐amyloid in at least four of eight regions, including the hippocampus, entorhinal cortex, midfrontal cortex, inferior temporal, angular gyrus, calcarine cortex, anterior cingulate cortex, and superior frontal cortex (Bennett et al., 2018). Tau tangle was a neurofibrillary tangle burden summary determined by microscopic examination of silver‐stained slides from five regions: midfrontal cortex, midtemporal cortex, inferior parietal cortex, entorhinal cortex, and hippocampus. Its value was calculated by the average count of each region divided by the corresponding standard deviation. Tangle density was the average of the cortical density (per mm^2^), which was determined using systematic sampling in at least four of eight regions as above.

### 2.6 Statistical analysis

To identify the optimal DNAm clocks capturing AD's neuropathological traits, we performed multiple linear regression models to examine the associations between 13 DNAm clocks and AD's neuropathological traits (i.e., β‐amyloid, Tau tangles, and tangle density). Chronological age, sex, PMI, study (ROS or MAP), and proportion of neurons in nervous tissue (prop.N) were considered as covariates. Especially, prop.N was calculated using the CETS algorithm. Using principal component analysis (PCA), we also calculated the first principal component (PC1) of selected DNAm clocks as a composite aging measure.

To identify the key gene modules associated with AD's neuropathological traits, first, WGCNA based on RNA‐seq was introduced to cluster genes into gene modules and obtain the first component (ME) of every gene module, and the Student asymptotic *p*‐values were recorded (Horvath, 2011). Second, correlation analysis via WGCNA was conducted to identify the key gene modules (presented by ME) associated with both AD's neuropathological traits and aging (in other words, the significant AD‐related DNAm clocks). Furthermore, the top 20 genes with the largest intramodular connectivity and significant *p*‐value were defined as the hub genes of one module.

To further validate the associations of hub genes with DNAm clocks and AD, the proteins coded by hub genes were identified as hub proteins given the hereditary information flow in the Central Dogma. Linear regression with adjustment for age, sex, PMI, study, and prop.N was applied to examine the associations between the abundance of hub proteins with DNAm clocks and neuropathological traits of AD. Then, the hub proteins associated with both DNAm clocks and neuropathological traits were employed in the further validation at the RNA‐seq level with the diagnosis of AD among the ROSMAP and three independent datasets (i.e., GSE5281 (Liang et al., 2007) with 85 AD and 73 control samples, GSE118553 (Patel et al., 2019) with 167 AD and 98 control samples, and GSE1297 (Blalock et al., 2004) with 22 AD and 9 control samples). Moreover, we used regression model for ordered data (plor() function in R) to examine the associations of the selected key gene modules, genes and proteins with the clinical consensus diagnosis of cognitive status at the time of death, which is a hallmark of AD.

All the statistical analyses were conducted using R version 4.0.3. For all tests, two‐tailed *p* < 0.05 was adopted as statistically significant and the Bonferroni method (Sun et al., 2020) was used for multiple testing correction.

## 3 RESULTS

### 3.1 Associations of DNAm clocks with AD's neuropathological traits

**FIGURE 2:**
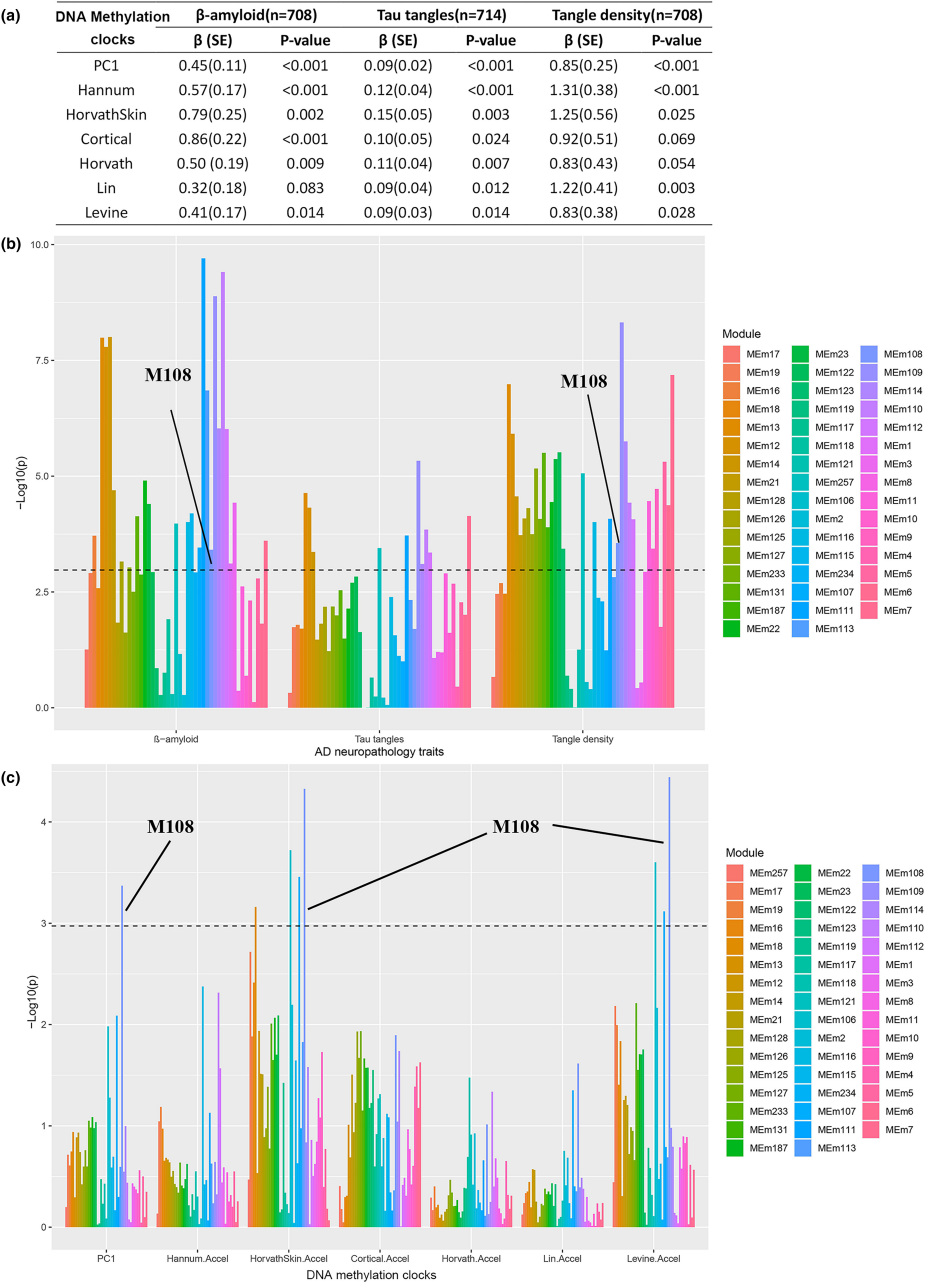
Associations among neuropathological traits of Alzheimer's disease, DNA methylation, and RNA‐seq. SE, standard error. (a) The associations of selected DNA methylation clocks with β‐ amyloid protein level, neurofibrillary tangle summary, and tangle density. The number of participants varied slightly for different outcomes in the Table, due to the partial lack of neuropathological traits. The models were adjusted for chronological age, sex, cohort study, postmortem interval, and the proportion of nerve cells. (b, c) The associations of selected gene modules with common Alzheimer's disease‐related neuropathological traits (b) and DNA methylation clocks (c). Results are presented as −log_10_(*p*‐value), the higher the bar graph, the more significant the associations. The horizontal dashed line is the significant line after Bonferroni correction (number of tests = 47).

All DNAm clocks, except for GrimAge, Yang, and Bocklangt clocks, were found to be positively associated with AD neuropathological traits. For instance, the Hannum clock was positively associated with all β‐amyloid (*β* = 0.57, SE = 0.17, *p* < 0.001), Tau tangles (*β* = 0.12, SE = 0.04, *p* < 0.001), and tangle density (*β* = 1.31, SE = 0.38, *p* < 0.001) (Figure 2a). Then six DNAm clocks (i.e., Hannum, HarvathSkin, Cortical, Horvath, Lin, and Levine clocks) associated with at least two neuropathological traits were included in the following analyses and were integrated as a new composite aging measure, namely, PC1 (explained 57.9% of the variances of six DNAm clocks, Figure S1). In addition, we observed that PC1 was positively associated with β‐amyloid (*β* = 0.45, SE = 0.11, *p* < 0.001), Tau tangles (*β* = 0.09, SE = 0.02, *p* < 0.001) and tangle density (β = 0.85, SE = 0.25, *p* < 0.001). The details are listed in Table S1.

### 3.2 Association between gene modules and AD neuropathological traits

Of 11,161 genes obtained from ROSMAP, 47 modules were identified via WGCNA by Mostafavi et al. (2018). Based on the PC1 of each module, we found that ten gene modules (i.e., m13, m12, m14, m257, m111, m109, m114, m110, m112, and m7) were associated with all three AD neuropathological traits after Bonferroni correction (number of tests = 47). For instance, m109 was correlated with β‐amyloid, Tau tangles, and tangle density significantly (biweight midcorrelations = 0.27, 0.20, and 0.26, respectively, and *p* = 1.30 × 10^−9^, 4.65 × 10^−6^, and 4.79 × 10^−9^, respectively). Besides, eight gene modules (i.e., m21, m126, m127, m131, m22, m23, m116, and m108) were associated with both β‐amyloid and tangle density (Figure 2b).

### 3.3 Association between gene modules and DNAm clocks

After Bonferroni correction (number of tests = 47), gene modules m18, m106, m107, and m108 were associated with HorvathSkin clock AA; while m106, m107, and m108 were associated with Levine clock AA. For example, the biweight midcorrelations of m108 associated with HorvathSkin and Levine clock AA were both −0.18 (*p* = 4.70 × 10^−5^ and 3.61 × 10^−5^, respectively). In particular, module m108 was significantly associated with PC1 of six AAs (*p* < 0.001, Figure 2c).

### 3.4 Identifying hub genes in gene module m108

Synthesizing the findings above, gene module m108 which was statistically significantly associated with both AD neuropathology and epigenetic aging was further analyzed. To detect the most important genes in m108, we defined the top 20 of 263 total genes (i.e., *HEPACAM*, *ARHGEF26*, *METTL7A*, *PBXIP1*, *SNTA1*, *SLC25A18*, *MLC1*, *SZRD1*, *KANK1*, *GRAMD1C*, *FAM107A*, *GPAM*, *SOX2*, *TNS3*, *CABLES1*, *CHDH*, *EYA2*, *MYO10*, *PAX6*, and *STON2*) with the largest intramodular connectivity (from 39.86 to 29.44) as the hub genes of m108. The details on the connectivity of the top 20 in m108 are listed in Table S2.

### 3.5 Proteins coded by hub genes of m108

**FIGURE 3:**
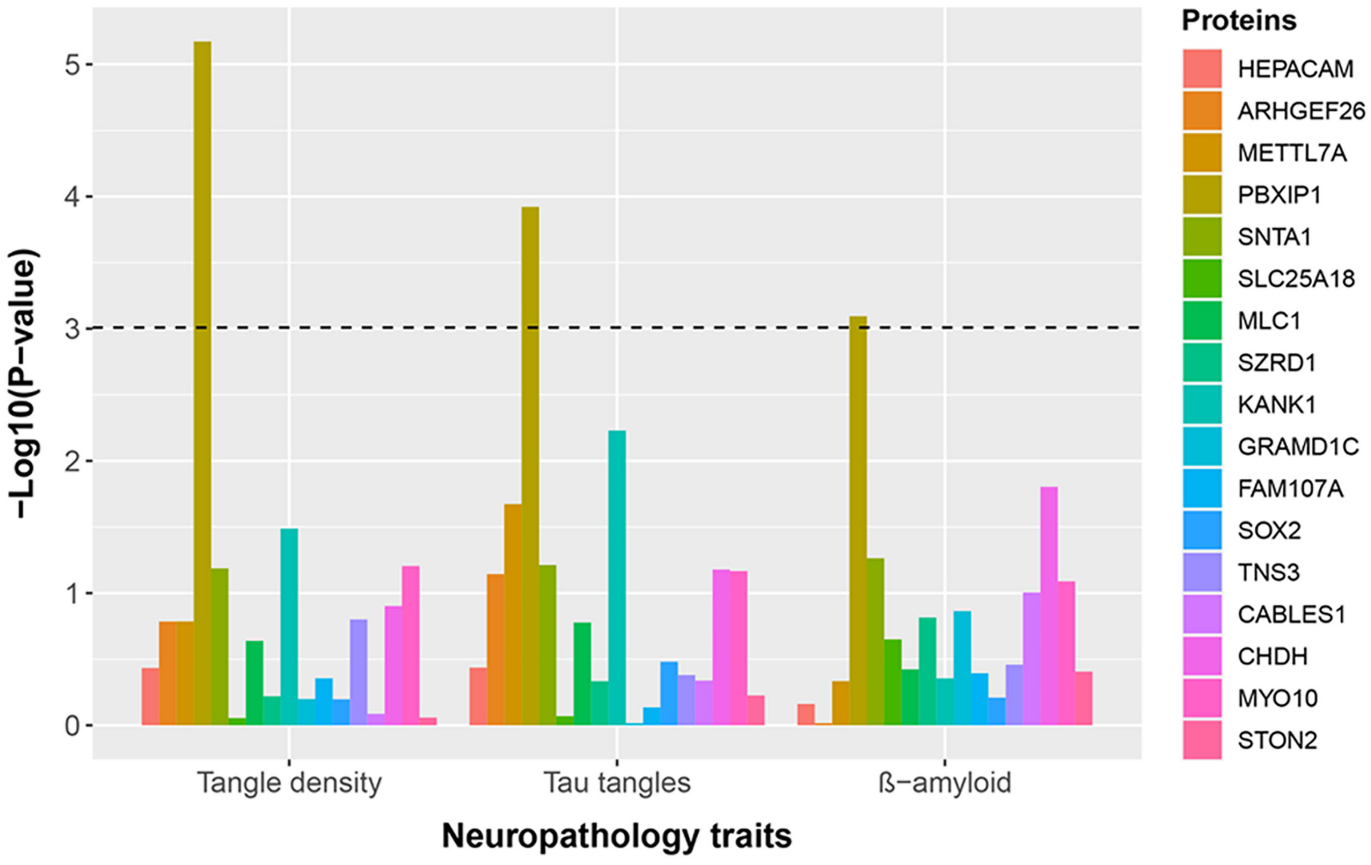
Association between the abundance of hub proteins and neuropathological traits of Alzheimer's disease. The horizontal dashed line is the significant line after Bonferroni correction (number of tests = 51).

Among 20 hub genes of m108, proteins coded by three genes (*GPAM*, *EYA2*, and *PAX6*) were not available in TMT proteomics data. Therefore, associations between the abundance of 17 coded proteins and neuropathological traits of AD were explored. As shown in Figure 3 and Table S3, the protein coded by *PBXIP1* was the only protein that was significantly associated with all three neuropathological traits of AD after adjusting for covariates (*β* = 5.97, 8.74, and 1.10 for β‐amyloid, Tau tangles, and tangle density, respectively, all *p* < 0.001). And proteins coded by *SNTA1*, *KANK1*, *CHDH*, and *MYO10* were also marginally significantly associated with two or three neuropathological traits of AD.

**FIGURE 4:**
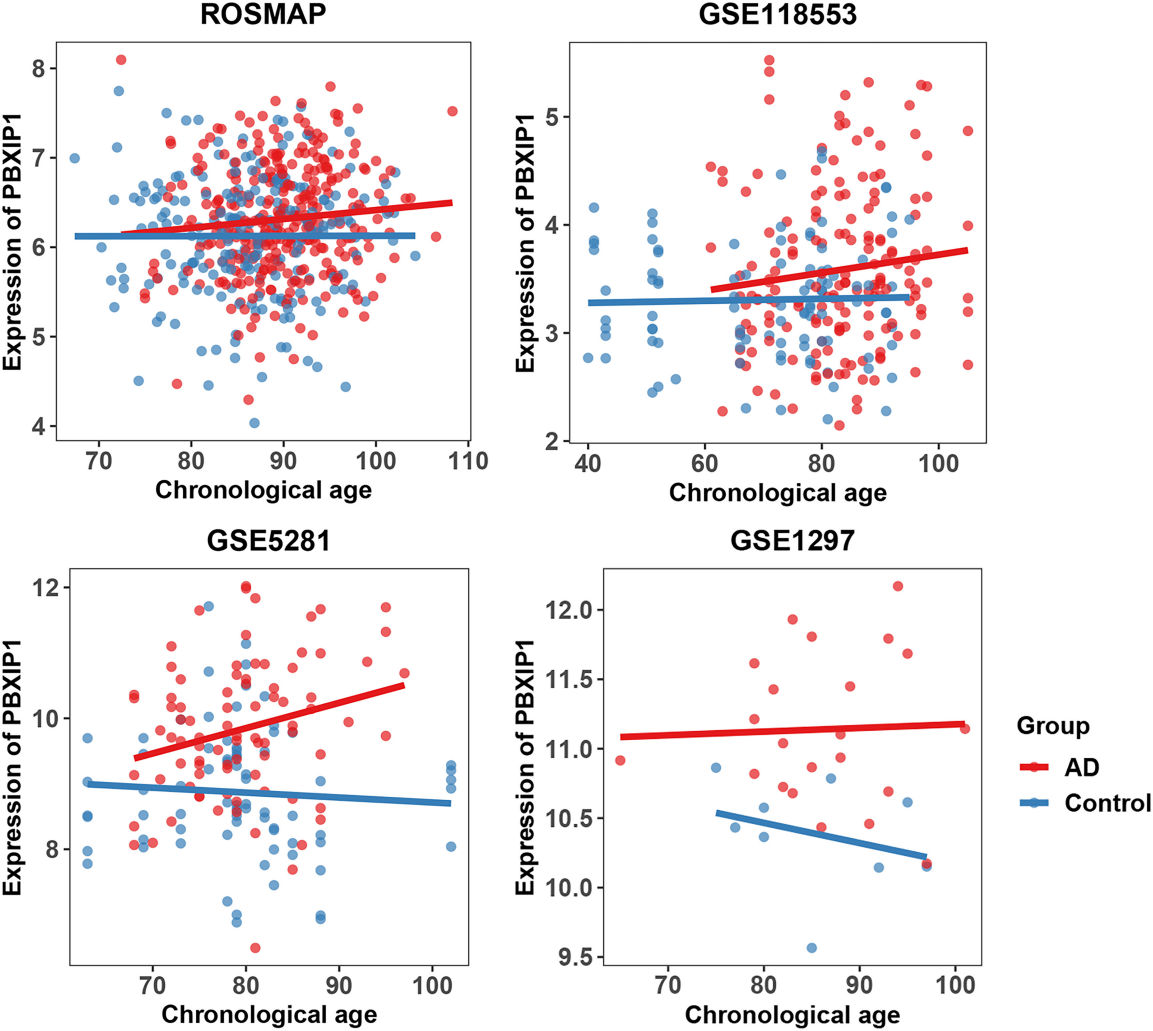
Expression of *PBXIP1* with chronological age by the diagnosis of Alzheimer's disease. AD, Alzheimer's disease; Control, participants without AD; ROSMAP, the Religious Orders Study and Rush Memory and Aging Project.

For further validation, we used linear regression models to explore the association of the expression of *PBXIP1* (at a single gene level rather than a module level) with DNAm clocks and neuropathological traits of AD (Figure S2). The results showed that the higher expression of *PBXIP1* was statistically significantly associated with larger tangle density (*β* = 1.00, SE = 0.24, *p* < 0.001). Moreover, the expression level of *PBXIP1* was clearly distinguished by the diagnosis of AD, and the trend of expression level changes with age in different datasets was consistent (Figure 4). In addition, the gene expression level of *PBXIP1* was associated with clinical diagnosis of AD across the datasets (odds ratio = 0.53, 95% confidence interval = 0.43–0.64, adjusted for age and the study using logistic regression). Moreover, gene module m108 and PBXIP1 (at both gene and protein levels) were associated with the clinical consensus diagnosis of cognitive status at time of death (Table S4).

## 4 DISCUSSION

Based on the reported 13 DNAm clocks and neuropathological data from ROSMAP, we identified six AD‐related DNAm clocks (i.e., Hannum, HorvathSkin, Cortical, Horvath, Lin, and Levine clocks). Moreover, the composite aging measure (PC1) based on these six clocks was associated with all three AD's neuropathological traits significantly. Then, based on RNA‐seq data, module m108 was identified as the key module associated with both DNAm clocks and AD's neuropathological traits using WGCNA. Among 20 key genes with top intramodular connectivity in m108, *PBXIP1* was found to be significantly associated with AD's neuropathological traits and aging at the proteomic level after multi‐comparison correction. Then the association between *PBXIP1* and the diagnosis of AD was validated in ROSMAP and another three independent datasets at the transcriptomics level.

Associations of several individual DNAm clocks with the diagnosis or pathological traits of AD have been explored in previous studies. For instance, Levine et al. (2015) reported that Horvath clock acceleration was positively associated with several neuropathological measurements including diffuse plaques, neuritic plaques, and amyloid load among individuals with AD, and then extended these associations to Levine clock. McCartney et al. (2018) found that intrinsic AA based on the Horvath clock and extrinsic AA based on the Hannum clock were both related to AD risk factors (for instance, body mass index, total cholesterol to high‐density lipoprotein cholesterol ratios, socioeconomic status, high blood pressure, and smoking behavior) after Bonferroni correction. However, our study extended these associations to more comprehensive DNAm clocks such as HorvathSkin and Lin clocks. More importantly, by using PCA, we found that the PC1 based on six DNAm clocks was positively associated with all three neuropathological traits of AD after multi‐comparison correction.

The consistent associations between *PBXIP1* (*PBX* homeobox interacting protein 1, also known as *HPIP*) at both mRNA and protein levels with AD might illustrate that *PBXIP1* gene performs biological functions on AD via the protein it coded according to Central Dogma. Although *PBXIP1* has been reported to be related to diverse biological processes such as hematopoiesis, estrogen signaling‐related cellular function, reproduction biology, and cancers (Khumukcham & Manavathi, 2021) since its discovery in 2000 (Abramovich et al., 2000), the role of *PBXIP1* on AD is rarely studied. For instance, via Harmonizome, a collection of information about genes and proteins from 114 datasets provided by 66 online resources (https://maayanlab.cloud/Harmonizome/), *PBXIP1* is reported to express increasingly in AD based on GSE1297 (a GEO dataset) with 8 control and 22 AD subjects. However, it is not related to the neurofibrillary tangle in GSE1297 (Blalock et al., 2004). Another study based on transcriptome data of 57 non‐dementia and 50 dementia participants from the Adult Change in Thought cohort reported that the higher expression level of *PBXIP1* is associated with the higher level of P‐Tau and Aβ40 in the tissue from temporal, even though the association of *PBXIP1* with P‐Tau is insignificant after adjusted for multi‐comparison (Ferrucci et al., 2018).

A probable explanation of the association between *PBXIP1* and AD is related to the roles of *PBXIP1* on astrocytes and hippocampal neurons (Seyfried et al., 2017; Sharma et al., 2015). *PBXIP1* is reported as an astroglial progenitor cell marker during human embryonic development (van Vuurden et al., 2014) as well as a cell‐type marker of astrocytes based on the transcriptome of the mouse (Sharma et al., 2015), and is included in a proteomics module enriched in astrocyte via WGCNA (Seyfried et al., 2017). Moreover, *PBXIP1* is a novel protein that is expressed in hippocampal neurons, and its expression is linked to neuronal degeneration in postmenopausal women (Karamese et al., 2013). Another mechanism might be related to the inhibition of mTOR through the ERK pathway. Previous studies have reported that down‐regulation of *PBXIP1* led to the repression of ERK and subsequent inhibition of mTOR (Xu et al., 2013). Meanwhile, a high prevalence of brain dysfunction disorders (including cognitive and behavioral impairments) has been reported to be associated with disturbances in ERK and mTOR signaling pathways (Krab et al., 2008). The above evidence supports the potential role of *PBXIP1* in the pathophysiologic process of AD.

### 4.1 Strengths and limitations

There are several strengths of our study. First, ROSMAP provides unique data across multi‐omics (i.e., epigenetics, transcriptomics, and proteomics) and the neuropathology of AD. Second, we studied the relationship with DNAm clocks via multi‐clocks and composite clocks of them rather than a single one; therefore, grasping more features of biological aging. Third, we implied three neuropathological traits rather than the clinical diagnosis of AD to better capture the pathophysiologic mechanism of AD. Furthermore, the association we identified was validated in several independent datasets. However, there are still several limitations in this study. First, the DNAm clock is only one biological hallmark of aging, which cannot explain the whole information about aging. Second, a clear difference between RNA‐seq and proteomic analyses is the sensitivity of these approaches. In addition, the association was not validated in vivo. However, for the heterogeneity of AD between humans and animal models, the consistency of validation in vivo is controversial. At last, short RNAs (such as microRNA) were not included although they are critical to the biological process of AD (Chen et al., 2021; Yin et al., 2023) given that we primarily concentrated on the hereditary information flow in the central dogma.

## 5 CONCLUSION

In summary, based on data cross multi‐omics (i.e., epigenetics, transcriptomics, and proteomics) and neuropathological traits of AD from the ROSMAP study, we identified a gene module (i.e., m108) related to both aging (DNAm clocks) and AD. Moreover, we validated *PBXIP1* in m108 was associated with AD's neuropathology at the proteomic level and AD's diagnosis in ROSMAP and another three independent datasets. This robust association supported the potential and feasibility of using multi‐omics data to investigate complex diseases and implied that *PBXIP1* and related pathways might explain the mechanism of AD. However, more validation in different populations and experiments in vitro and in vivo should be considered in the future.

## AUTHOR CONTRIBUTIONS

Zuyun Liu contributed to the conception and design of the work. Jingyun Zhang and Xiaoyi Sun performed the analysis. Jingyun Zhang and Xiaoyi Sun wrote the initial draft of the manuscript. Jingyun Zhang, Xiaoyi Sun, Xueqing Jia, Binggui Sun, Shijun Xu, Weiping Zhang, and Zuyun Liu contributed to the interpretation of data. Xueqing Jia, Binggui Sun, Shijun Xu, Weiping Zhang, and Zuyun Liu revised it critically for important intellectual content. All authors approved the final version of the manuscript. Zuyun Liu is the guarantor. The corresponding author attests that all listed authors meet authorship criteria and that no others meeting the criteria have been omitted.

## FUNDING INFORMATION

This work was supported by the Natural Science Foundation of Zhejiang Province (LQ21H260003), Research Center of Prevention and Treatment of Senescence Syndrome, School of Medicine Zhejiang University (2022010002), National Natural Science Foundation of China (82171584), “Pioneer” and “Leading Goose” R&D Programs of Zhejiang Province (2023C03163), and Key Laboratory of Intelligent Preventive Medicine of Zhejiang Province (2020E10004).

## CONFLICT OF INTEREST STATEMENT

The authors have no conflict of interest to declare.

## Supporting information


Data S1:

## Data Availability

This study was based on the public data from the ROSMAP, which could be applied from the official website (https:// www.radc.rush.edu). This study used data across multi‐omics (i.e., DNA methylation, RNA‐seq, proteomics, based on frozen tissue from DLPFC donated by ROSMAP participants after death) and neuropathological traits of 761 participants. All data were obtained through the AMP‐AD Knowledge Portal and Rush Alzheimer's Disease Center of Rush University (https://adknowledgeportal.synapse.org/Explore/Programs/DetailsPage?Program=AMP‐AD).
