# Supplementary material for: Integrative multi‐omics analysis reveals the critical role of the PBXIP1 gene in Alzheimer's disease
Source: Aging Cell. 2023 Nov 20;23(2):e14044. doi: 10.1111/acel.14044 (PMC10861197; doi:10.1111/acel.14044)
Supplement: Supplementary file 1 — Data S1: [file ACEL-23-e14044-s001.docx]

**Integrative multi-omics analysis reveals the critical role of the *PBXIP1* gene in Alzheimer's disease**

Jingyun Zhang ^1#^, Xiaoyi Sun ^1#^, Xueqing Jia ^1^, Binggui Sun ^2^, Shijun Xu ^3^,Weiping Zhang ^2^, Zuyun Liu ^1*^

^1^ Center for Clinical Big Data and Analytics of the Second Affiliated Hospital and Department of Big Data in Health Science School of Public Health, the Key Laboratory of Intelligent Preventive Medicine of Zhejiang Province, Zhejiang University School of Medicine, Hangzhou 310058, Zhejiang, China.

^2^ Department of Neurobiology, School of Basic Medical Sciences, Key Laboratory of Medical Neurobiology (Ministry of Health of China), Key Laboratory of Neurobiology of Zhejiang Province, Zhejiang University School of Medicine, Hangzhou 310058, Zhejiang, China.

^3^ Institute of Material Medica Integration and Transformation for Brain Disorders, and School of Pharmacy, Chengdu University of Traditional Chinese Medicine, Chengdu 611137, Sichuan, China.

^4^ Department of Pharmacology, Institute of Neuroscience, Key Laboratory of Medical Neurobiology of the Ministry of Health of China, Zhejiang Province Key Laboratory of Mental Disorder's Management, Zhejiang University School of Medicine, Hangzhou 310058, Zhejiang, China.

^#^ These two authors have contributed equally to this work.

* Corresponding author:

Zuyun Liu, Ph.D., Center for Clinical Big Data and Analytics of the Second Affiliated Hospital and Department of Big Data in Health Science School of Public Health, the Key Laboratory of Intelligent Preventive Medicine of Zhejiang Province, Zhejiang University School of Medicine, 866 Yuhangtang Rd, Hangzhou, 310058, Zhejiang, China. Telephone: +86-0571-87077127. Email: Zuyun.liu@outlook.com or zuyunliu@zju.edu.cn

**
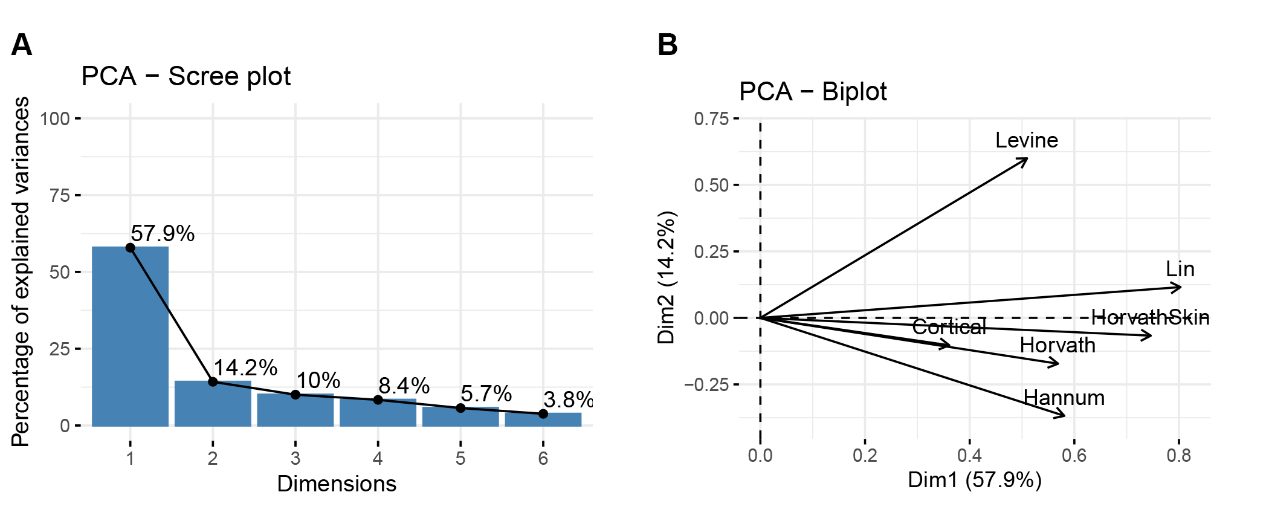
**

**Figure S1. The results of principle component analysis of six DNA methylation clocks.**

Notes: (A) is the scree plot, shows the percentage of explained variances of six principle components; (B) is the biplot, show the loading of six DNA methylation clocks on the first and second principle components.


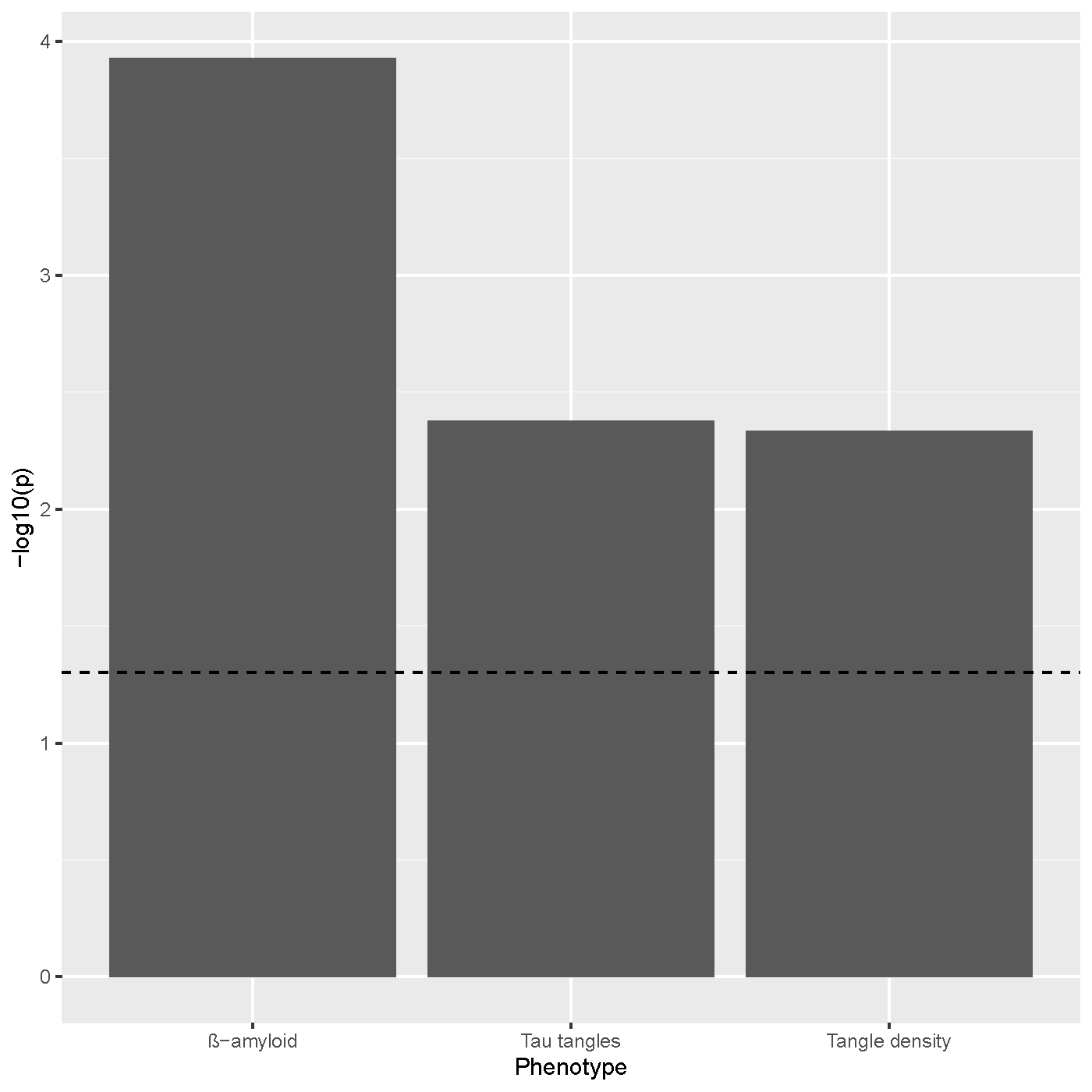


**Figure S2. The association of PBXIP1 with β-amyloid protein level, neurofibrillary tangle summary, and tangle density**

Notes: The models were adjusted for age, sex, cohort study, the proportion of nerve cells, and post-mortem interval. The horizontal dashed line is the reference cut-off, where p-value=0.05.

**Table S1. The associations of epigenetic clocks with β-amyloid protein level, Tau tangles, and tangle density**

| **Epigenetic clock** | **β-amyloid(n=708)** | | **Tau tangles(n=714)** | | **Tangle density(n=708)** | |
| --- | --- | --- | --- | --- | --- | --- |
|  | **β (SE)** | **p-value** | **β (SE)** | **p-value** | **β (SE)** | **p-value** |
| PC1 | 0.45(0.11) | <0.001 | 0.09(0.02) | <0.001 | 0.85(0.25) | <0.001 |
| Hannum | 0.57(0.17) | <0.001 | 0.12(0.04) | <0.001 | 1.31(0.38) | <0.001 |
| HorvathSkin | 0.79(0.25) | 0.002 | 0.15(0.05) | 0.003 | 1.25(0.56) | 0.025 |
| Cortical | 0.86(0.22) | <0.001 | 0.10(0.05) | 0.024 | 0.92(0.51) | 0.069 |
| Horvath | 0.50(0.19) | 0.009 | 0.11(0.04) | 0.007 | 0.83(0.43) | 0.054 |
| Lin | 0.32(0.18) | 0.083 | 0.09(0.04) | 0.012 | 1.22(0.41) | 0.003 |
| Levine | 0.41(0.17) | 0.014 | 0.09(0.03) | 0.014 | 0.83(0.38) | 0.028 |
| Grimage | 0.29(0.38) | 0.446 | -0.07(0.08) | 0.353 | -0.44(0.85) | 0.599 |
| Weidner | 0.27(0.16) | 0.087 | 0.05(0.03) | 0.138 | 0.38(0.36) | 0.280 |
| VidalBralo | 0.13(0.15) | 0.363 | 0.03(0.03) | 0.396 | 0.11(0.33) | 0.734 |
| Zhang | 0.07(0.14) | 0.559 | 0.00(0.03) | 0.903 | 0.07(0.32) | 0.833 |
| Yang | -0.15(0.14) | 0.278 | -0.02(0.03) | 0.448 | -0.25(0.31) | 0.421 |
| Bocklandt | -0.41(0.14) | 0.003 | -0.06(0.03) | 0.034 | -0.78(0.32) | 0.015 |
| Garagnani | 0.45(0.18) | 0.012 | 0.06(0.04) | 0.085 | 0.72(0.40) | 0.073 |

Notes: SE, standard error. The number of participants varied slightly for different outcomes in the Table, due to the partial lack of neuropathology traits. The models were adjusted for age, sex, cohort study, proportion of nerve cells, and Post-mortem interval.

**Table S2. Connectivity of top 20 genes in gene module m108**

| **Gene** | **kTotal** | **kWithin** | **kOut** | **kDiff** |
| --- | --- | --- | --- | --- |
| HEPACAM | 332.51 | 39.86 | 292.64 | -252.78 |
| ARHGEF26 | 462.36 | 36.25 | 426.11 | -389.85 |
| METTL7A | 459.87 | 35.03 | 424.84 | -389.81 |
| PBXIP1 | 257.07 | 34.44 | 222.63 | -188.19 |
| SNTA1 | 350.19 | 34.40 | 315.79 | -281.40 |
| SLC25A18 | 313.78 | 34.00 | 279.79 | -245.79 |
| MLC1 | 250.69 | 33.79 | 216.90 | -183.11 |
| SZRD1 | 713.95 | 33.00 | 680.94 | -647.94 |
| KANK1 | 427.16 | 32.34 | 394.82 | -362.48 |
| GRAMD1C | 248.71 | 32.09 | 216.62 | -184.53 |
| FAM107A | 431.21 | 30.87 | 400.34 | -369.46 |
| GPAM | 212.10 | 30.56 | 181.54 | -150.98 |
| SOX2 | 396.10 | 30.42 | 365.68 | -335.25 |
| TNS3 | 320.98 | 30.38 | 290.60 | -260.21 |
| CABLES1 | 201.12 | 30.28 | 170.85 | -140.57 |
| CHDH | 450.27 | 30.26 | 420.01 | -389.76 |
| EYA2 | 296.79 | 30.19 | 266.60 | -236.41 |
| MYO10 | 390.00 | 30.14 | 359.86 | -329.72 |
| PAX6 | 291.54 | 29.79 | 261.75 | -231.96 |
| STON2 | 323.33 | 29.44 | 293.89 | -264.45 |

Notes: KTotal, total connectivity of the gene, the sum of KWithin and KOut; KWithin, intramodular connectivity of the gene; KOut, connectivity outside the module of the gene; KDiff, difference between KWithin and KOut.

**Table S3. The associations of hub proteins in m108 with overall amyloid level, Tau tangles, and tangle density**

| **Proteins** | **β-amyloid** | | **Tau tangles** | | **Tangle density** | |
| --- | --- | --- | --- | --- | --- | --- |
|  | **β (SE)** | **p-value** | **β (SE)** | **p-value** | **β (SE)** | **p-value** |
| ARHGEF26 | 0.10(2.31) | 0.964 | 5.31(2.93) | 0.072 | 0.45(0.32) | 0.164 |
| CABLES1 | 4.68(2.82) | 0.099 | -2.70(3.64) | 0.459 | 0.09(0.40) | 0.82 |
| CHDH | 3.17(1.30) | 0.016 | 3.10(1.68) | 0.066 | 0.28(0.18) | 0.125 |
| FAM107A | 0.69(0.82) | 0.404 | 0.36(1.06) | 0.732 | 0.09(0.11) | 0.441 |
| GRAMD1C | -2.37(1.59) | 0.137 | -0.09(2.04) | 0.965 | 0.11(0.22) | 0.633 |
| HEPACAM | 0.33(0.83) | 0.69 | 0.96(1.06) | 0.366 | 0.10(0.12) | 0.369 |
| KANK1 | 1.72(2.24) | 0.442 | 7.84(2.82) | 0.006 | 0.66(0.31) | 0.033 |
| METTL7A | 1.54(2.10) | 0.464 | 6.16(2.65) | 0.021 | 0.41(0.29) | 0.164 |
| MLC1 | 1.40(1.58) | 0.378 | 2.81(2.03) | 0.167 | 0.27(0.22) | 0.23 |
| MYO10 | -2.54(1.45) | 0.082 | 3.41(1.86) | 0.068 | 0.38(0.2) | 0.062 |
| PBXIP1 | 5.97(1.75) | 0.001 | 8.74(2.23) | <0.001 | 1.10(0.24) | <0.001 |
| SLC25A18 | -2.31(1.90) | 0.224 | -0.45(2.44) | 0.853 | -0.04(0.27) | 0.882 |
| SNTA1 | 4.86(2.51) | 0.055 | 6.06(3.22) | 0.061 | 0.65(0.35) | 0.065 |
| SOX2 | 1.06(2.13) | 0.618 | 2.66(2.73) | 0.331 | 0.14(0.30) | 0.636 |
| STON2 | 1.17(1.36) | 0.392 | -0.93(1.75) | 0.595 | 0.03(0.19) | 0.874 |
| SZRD1 | 3.97(2.77) | 0.153 | 2.61(3.56) | 0.464 | 0.20(0.39) | 0.604 |
| TNS3 | 1.75(1.86) | 0.348 | 1.94(2.39) | 0.417 | 0.37(0.26) | 0.158 |

Notes: SE, standard error.

**Table S4. Associations of gene model m108 and PBXIP1 with cogdx**

| **Association between gene module m108 and cogdx** | | | |
| --- | --- | --- | --- |
|  | **Correlation ^1^** | **P-value** | **Significance after Bonferroni correction** |
| m108 | 0.16 | 3.1 × 10^-4^ | + |
| **Association between PBXIP1 and cogdx** | | | |
|  | **HR (95% CI)** | **P-value** | **Significance after Bonferroni correction** |
| Gene | 1.5 (1.2, 2.0) | 1.3 × 10^-3^ | + |
| Protein | 1.8 (1.3, 2.4) | 1.3 × 10^-3^ | + |

Notes: HR=hazard ration; CI=confidence interval.

^1^ The correlation was calculated using function bicor() in Package WGCNA (R language).
